# Supplementary material for: Complexome profiling of the Chlamydomonas psb28 mutant reveals TEF5 as an early PSII assembly factor
Source: Plant Cell. 2025 Mar 17;37(6):koaf055. doi: 10.1093/plcell/koaf055 (PMC12164590; doi:10.1093/plcell/koaf055)
Supplement: koaf055_Supplementary_Data [file koaf055_supplementary_data.zip › psb28_interactive.zip!/plots/intensity/qualityPlots/peptides/CPL23_Cre02.g141100.t1.2.html]

### Cre02.g141100.t1.2

Trivial name: CPL23  
SeqCoverage [median]: 21.80  
Mass: 42.09 kDa  
  
 

| Sequence | MissedCleavage | Mass [Da] | wtIntensity | muIntensity | Score |
| --- | --- | --- | --- | --- | --- |
| AAMSNSPLK | 0 | 917.46 | 0.00 | 395.99 | 131.62 |
| AAMSNSPLKK | 1 | 1045.56 | 4060.43 | 3879.69 | 72.65 |
| FVNNVIGGENFIDMAR | 0 | 1794.87 | 0.00 | 9523.24 | 136.70 |
| ILADPLFLFK | 0 | 1175.70 | 2930.50 | 14680.38 | 143.01 |
| KLSLPADMLEVAKK | 2 | 1541.89 | 0.00 | 1043.42 | 46.41 |
| LSLPADMLEVAK | 0 | 1285.70 | 0.00 | 1642.24 | 71.35 |
| LSLPADMLEVAKK | 1 | 1413.79 | 924.73 | 630.85 | 51.21 |
| LVDMTIAR | 0 | 917.50 | 3047.41 | 11555.82 | 157.58 |
| SIPQVAYGTTIAIR | 0 | 1488.83 | 1876.83 | 6643.39 | 134.98 |
| TFNVGQR | 0 | 820.42 | 739.68 | 2271.35 | 92.69 |
| YQIVFGLER | 0 | 1123.60 | 1289.32 | 0.00 | 90.76 |
|  |  |  | 14868.9 | 52266.4 |  |
